# Serum Therapeutics at the British Medical Association

**Published:** 1895-08-24

**Authors:** 


The Hospital

AN INSTITUTIONAL JOURNAL OF

The Medical Sciences and Hospital Administration,

WITH

AN EXTRA NURSING SUPPLEMENT.

VOL. XVIII.—No. 465.] [AUG. 24, 1895.

### SERUM THERAPEUTICS AT THE BRITISH MEDICAL ASSOCIATION.

It has been said that medicine at its circumference is always half a century behind medicine at its centre. If that be so it is manifestly a matter of great moment, both to the profession and to those who avail themselves of its services, that some method be found of reducing this period within limits which more approximate to scientific reason. The discussion on "Serum Therapeutics" which occupied so prominent a place at the recent meetings of the British Medical Association showed to us one method by which this can be done.

A concentration of competent observation and experience was the most marked feature of the discussion. Nobody who was present at the sectional meetings, or who will take the trouble to read the detailed reports thereupon, can be in any doubt as to the thoroughness and competency of the investigation to which serum therapeutics have been subjected, especially in the case of diphtheria, in our cautious country. This is as it should be. Incompetent observers and hasty generalisers are among the most dangerous enemies that medical science has now and has always had to grapple with. The discussion on serum therapeutics in its application to diphtheria at the British Medical Congress was in every respect a serious contribution to practical therapeutics, and worthy of the best traditions of British medicine.

There emerges as the net result two demonstrations. The first is that the serum of animals is a powerful physiological agent, and that natural serum may be so modified as to produce marked physiological effects of a beneficial character upon other animals into whose bodies it may be introduced. Dr. Klein's learned and convincing arguments, constituting as they do the plain and unvarnished exposition of his scientific processes and results in the laboratory, leave no doubt whatever upon the candid mind as to the physiological potency of modified and unmodified animal serum. Here, in a word, is an agent of mighty powers. If space permitted it would be easy to show, *a priori*, that such a fact is exactly what we might have anticipated. From the earliest times the world has been familiar with the wonderful potencies stored up in the fluids and solids of the vegetable kingdom. Why should it be thought incredible, or even strange, that equally or even more remarkable properties should be found stored up in the more highly organised structures of the animal world? Serum, we repeat, for good or for ill, is a powerful agent. It may prove, in no long time, an agent which will revolutionise the whole of medical practice.

The second positive demonstration which emerges from the recent discussion is that serum anti-toxin has, without any doubt whatever, a marked effect upon patients suffering from diphtheria, and upon the local manifestations of the diphtheritic state. So convinced is Dr. Washbourn on this point that he does not hesitate to declare that the "discovery of serum therapeutics marks a distinct epoch in the history of medicine." What the practical physician who carries his conscience to the bedside wishes to know is this: Does the anti-toxin treatment favourably modify the course of an attack of diphtheria? does it lessen mortality from this disease? is it accompanied by any circumstances of a seriously injurious kind? In Dr. Washbourn's judgment, and he was supported by practically every other speaker at the meeting, anti-toxin modifies diphtheria favourably, more favourably than any other known method of treatment; and it lowers mortality by as much as 25 per cent. Moreover, injuries of a serious kind, which have been stated to result from its use, are found to be on investigation not only not proved but disproved.

It seems then that a third result ought to emerge from this concentration of laboratory results and competent clinical experience at the Annual Congress of British Medicine. That result should be nothing less than an unavoidable obligation operating throughout the whole length and breadth of the medical profession. Every medical man, whether in private or hospital practice, should as opportunity offers feel bound to try the anti-toxin treatment for diphtheria; and to test it conscientiously and thoroughly. It seems to us, after the convincing demonstrations of Dr. Klein in the laboratory and Dr. Washbourn and others at the bedside, that no medical man can with a good conscience refuse to give his diphtheritic patients the advantage of the new treatment.

It is worth while to note another point which the discussion has brought out with much distinctness, and that is the important part which hospitals have played in these investigations. So much has this been the case that we can all see that if there had been no hospitals available for examining this question on a large scale there would have been no adequate confirmation of the results of physiological laboratory work at all. The hospitals have furnished the two indispensable conditions of trustworthy investigation, namely, competent observers, and patients in sufficient numbers. It remains but to insist that serum therapy, as applied to diphtheria, has been subjected to every possible test but that of time; and that so far as present appearances are concerned it is the most effective treatment for that dangerous disease which scientific medicine as yet knows.

